# Why do plants need the ZMM crossover pathway? A snapshot of meiotic recombination from the perspective of interhomolog polymorphism

**DOI:** 10.1007/s00497-022-00446-3

**Published:** 2022-07-12

**Authors:** Piotr A. Ziolkowski

**Affiliations:** Laboratory of Genome Biology, Institute of Molecular Biology and Biotechnology, Adam Mickiewicz University, Poznań, Poland

## Abstract

At the heart of meiosis is crossover recombination, i.e., reciprocal exchange of chromosome fragments between parental genomes. Surprisingly, in most eukaryotes, including plants, several recombination pathways that can result in crossover event operate in parallel during meiosis. These pathways emerged independently in the course of evolution and perform separate functions, which directly translate into their roles in meiosis. The formation of one crossover per chromosome pair is required for proper chromosome segregation. This “obligate” crossover is ensured by the major crossover pathway in plants, and in many other eukaryotes, known as the ZMM pathway. The secondary pathways play important roles also in somatic cells and function mainly as repair mechanisms for DNA double-strand breaks (DSBs) not used for crossover formation. One of the consequences of the functional differences between ZMM and other DSB repair pathways is their distinct sensitivities to polymorphisms between homologous chromosomes. From a population genetics perspective, these differences may affect the maintenance of genetic variability. This might be of special importance when considering that a significant portion of plants uses inbreeding as a predominant reproductive strategy, which results in loss of interhomolog polymorphism. While we are still far from fully understanding the relationship between meiotic recombination pathways and genetic variation in populations, recent studies of crossovers in plants offer a new perspective.

## Introduction

Sexual reproduction requires meiotic division, which emerged at the dawn of eukaryotic evolution (Barton and Charlesworth 1998; Villeneuve and Hillers 2001). To meet this requirement, most species rely on crossovers to segregate their homologous chromosomes and halve the chromosomes content creating sexual cells. This phenomenon is both necessary for the proper segregation of chromosomes during the first meiotic division and crucial for mixing genetic material from both parents, thus contributing to the maintenance of genetic variation on the population scale (Villeneuve and Hillers 2001; Mercier et al. 2015). While homologous recombination already existed in prokaryotes and often accompanies repair of DNA damage in somatic cells, a specific recombination pathway with unique features is responsible for the formation of most crossovers during meiosis in plants and most studied eukaryotes (Villeneuve and Hillers 2001; Pyatnitskaya et al. 2019).

In this opinion, I will discuss how the different recombination pathways contribute to shaping genomes by shuffling parental alleles, and how that might have contributed to the evolutionary history of plant genome content and organization. I will also briefly discuss specific roles in recombination pathways in meiosis, with an emphasis on their response to the DNA polymorphism present between homologous chromosomes (so-called interhomolog polymorphism or heterozygosity). I will focus mainly on the ZMM- and MUS81-dependent pathways, as our knowledge of alternative routes for crossover generation in plants, including the recently proposed FANCD2 pathway, is limited (Kurzbauer et al. 2018; Li et al. 2021). Furthermore, I will examine the consequences of polymorphism-oriented recombination on the genetic variation in inbreeding plant populations.

## Initial stages of recombination

**Fig. 1:**
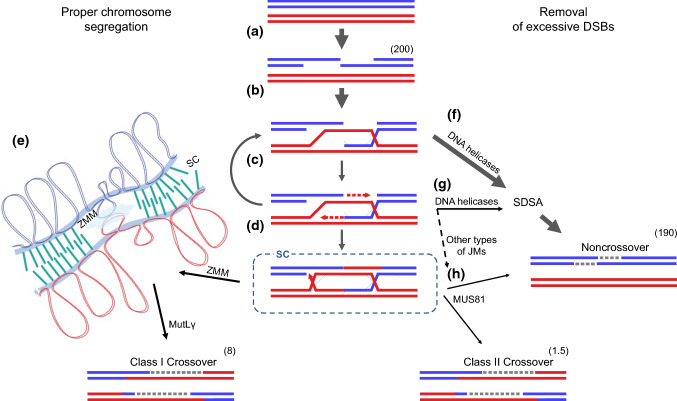
The ZMM and MUS81 recombination pathways have different functions in meiosis. Meiotic recombination starts with DSB formation and 5’-3’ DNA resection (**a**). 3’ single-stranded DNA invades the homologous chromatid and forms a D-loop (**b**). This can lead to DNA synthesis (dashed red arrows) and second-end capture, which results in dHJ formation (**c**, **d**). dHJs, when protected by ZMM proteins, will be converted to Class I crossovers by MutLγ resolvase. This normally takes place in the environment of the SC, which is involved in Class I crossover regulation (**e**). Hence, Class I crossovers serve to enable proper chromosome segregation during meiosis. DSBs that were not processed as crossovers are repaired by pathways leading to synthesis-dependent strand annealing (SDSA), which results in NCOs (**f**, **g**). In plants, conversion tracts (dashed gray lines) associated with NCOs are rarely observed, which suggests that DNA helicases (mainly RECQ4 and FANCM) displace invading strands at early stages or that strand extension by DNA polymerases is limited (**g**). The few JMs (including dHJs) that have escaped unraveling by helicases can eventually be resolved by MUS81 to produce either a crossover or an NCO (**h**). The numbers in brackets indicate approximate estimates of the frequency of each event per Arabidopsis meiosis. The arrow between (**d**) and (**c**) indicates recurrent rounds invasion, extension, and displacement resulting in complex structures; for simplicity, multiple conversion tracts are not shown on (**d**) and recombination outcomes

Before we can delve into the characteristics of the various recombination pathways, we need to briefly review the early stages of recombination that these pathways share. Recombination starts at the very beginning of meiosis with the formation of DNA double-strand breaks (DSBs) by the transesterase SPO11 (Keeney and Neale 2006; Vrielynck et al. 2021). Then, DSBs undergo resection to generate 3’-ssDNA ends (Fig. 1a). These ssDNA fragments are bound by the recombinases RAD51 and DMC1, which stimulate the key process in recombination, namely, strand invasion (Keeney and Neale 2006). Strand invasion may target the sister chromatid, which is generated by the replication of each chromosome preceding entry into meiosis (not shown in Fig. 1), or one of the non sister chromatids of the homologous chromosome (Fig. 1b) (Wang and Copenhaver 2018). The presence of the meiosis-specific recombinase DMC1 and chromosome axis components (see below) makes the homologous chromatid the preferred target in meiosis (so-called homolog bias) (Kim et al. 2010). From this point on, the two homologous chromosomes are linked by the invading DNA strand; hence, the site where they join is often referred to as a joint molecule (JM), regardless of the conformation adopted. As a result of the strand invasion step, a displacement loop (D-loop) is created by the extension of the invading strand by DNA polymerases (Fig. 1c). Multiple rounds of strand invasion, extension, and displacement often occur at this stage leading to complex recombination intermediates (Marsolier-Kergoat et al. 2018; Ahuja et al. 2021). The majority of these intermediates is then dissociated by DNA helicases, and the displaced strand is repaired by synthesis-dependent strand annealing (SDSA) (Fig. 1f, g). Alternatively, second-end capture can occur to form a double Holiday junction (dHJ) (Fig. 1d), which can be again displaced by helicases and subjected to SDSA repair (Fig. 1h) (Wang and Copenhaver 2018). In both cases, the resolution process leads to a non-crossover event (NCO), where only a very short patch of DNA, the fragment synthesized by polymerase using the invaded strand as a template, has been modified. JMs can also be resolved by specific nucleases, which can lead to either NCOs or crossovers (Fig. 1e, h) (Mercier et al. 2015).

Importantly, meiotic recombination usually occurs in the environment of the synaptonemal complex (SC), the protein structure that stabilizes pairing of homologous chromosomes to each other along their length (Fig. 1d, e). The SC is a tripartite structure assembled with two lateral elements constituting the meiotic chromosome axis, each anchoring the chromatids of one homologous chromosome, and a central element that spans the axes and contains the transverse element (Page and Hawley 2004). SC formation begins in zygotene by the progressive installation of numerous transverse elements between aligned homologs in a zipper-like manner (Cahoon and Hawley 2016). In a number of species, the transverse element is required for crossover formation via the ZMM pathway (Fig. 1e), providing a functional link between the SC and recombination (Pyatnitskaya et al. 2019).

### Multiple recombination pathways are required to ensure both genome stability and chromosome segregation during meiosis

Nearly all eukaryotes, including all plants studied thus far, have the same major meiotic crossover pathway—often referred to as the ZMM pathway (Villeneuve and Hillers 2001). This name comes from the first letters of the proteins required for this process in yeast: ZIP (ZIP1, ZIP2, ZIP3, and ZIP4), MER3, and MSH (MSH4 and MSH5) (Börner et al. 2004; Snowden et al. 2004). The ZMM proteins create an environment that stabilizes D-loops, which allows the formation of dHJs and the subsequent separation of JMs through the action of the meiosis-specific MLH1-MLH3 heterodimer (Pyatnitskaya et al. 2019). Interestingly, the MLH1-MLH3 complex preferentially resolves dHJs via crossover, the mechanism for which was recently deciphered in yeast (Cannavo et al. 2020; Kulkarni et al. 2020). Crossovers formed via the ZMM pathway are referred to as Class I crossovers, and their number and distribution along the chromosomes are tightly controlled. Each pair of homologous chromosomes experiences at least one crossover during meiosis (so-called obligate crossover or crossover assurance), but if more than one crossover occurs between chromosomes, the additional crossover events are further apart than would occur in a random distribution (so-called crossover interference) (Berchowitz and Copenhaver 2010; Dluzewska et al. 2018). In plants, Class I crossovers can be detected cytologically by the immunolocalization of some ZMM proteins in the pachytene/diakinesis stage, e.g., MLH1, MLH3, or HEI10 (the plant homolog of Zip3) (Chelysheva et al. 2010, 2012).

For a long time, it seemed that the SC was necessary for Class I crossovers in plants, but studies of plants bearing mutations in transverse filament-encoding genes (*ZEP1* and *ZYP1* in rice and Arabidopsis, respectively) have shown that this is not the case; in these mutants, the number of Class I crossovers (defined as MSH5-dependent and marked by HEI10/MLH1) increased, while crossover interference disappeared (Wang et al. 2010, 2015a; Capilla-Pérez et al. 2021; France et al. 2021). These mutants show only slightly reduced fertility caused by loss of crossover assurance. Therefore, it can be concluded that the primary role of the SC in plants is to support chromosome pairing in meiosis by bringing homologs into proximity (ca. 100 nm versus 400 nm without SC) and to control the frequency and chromosomal distribution of crossover events. Unlike mutants of genes involved in the formation of Class II crossovers including *MUS81* (Hartung et al. 2006), no phenotypes exceeding those observed in wild-type have been reported for most of the *ZMM* mutants in genotoxic stresses (Manova and Gruszka 2015; Pyatnitskaya et al. 2019). Thus, despite the separation of functions between the SC and crossover formation in plants, the ZMM pathway appears to be characteristic of only the repair of DSBs formed during meiosis prophase I.

In contrast, other DSB repair pathways are active also outside meiosis: both the SDSA pathway, whose products are exclusively NCOs, and MUS81-dependent resolution, which can result in either NCO or crossover (Fig. 1), rely on enzymes that are also important for the repair of DNA damage in somatic cells. Such damage occurs relatively often during DNA replication, as evidenced by the lethality that occurs when more than one repair pathway is turned off (Hartung et al. 2006; Crismani et al. 2012; Zhu et al. 2021). Disabling the DNA helicases FANCM or RECQ4 in plants results in a significant increase in crossover repair rates, suggesting that their role in meiosis is to repair SPO11-dependent DSBs via NCOs and remove intermediates that can be a substrate for MUS81-dependent crossover formation (Crismani et al. 2012; Séguéla-Arnaud et al. 2015). Therefore, it can be considered that additional recombination pathways in plants are mainly “cleaning up after SPO11”, but in a way that does not leave too many traces in the form of crossover (notably, the cleaning, in this case, is so effective that it is very difficult to detect even conversion tracts that would indicate NCOs; Fig. 1h). For reasons that are not entirely clear, the numbers of crossovers are generally kept low, between one and three per chromosome pair (Mercier et al. 2015). Since the ZMM pathway, due to its intrinsic interhomolog bias, would perform DSB repair preferentially by crossovers, additional pathways are required to repair DSBs via NCOs.

Alternatively, the same outcome could be achieved by reducing the number of DSBs generated during meiosis and performing repair exclusively via the ZMM pathway. The most likely reason why this is not happening in plants is another function of the meiotic DSBs that is facilitating homolog recognition during meiotic chromosome pairing (Zickler and Kleckner 2015). However, it should be noted that DSB-mediated pairing does not occur in all eukaryotes, though it is considered as canonical. Organisms that use alternative pairing strategies tend to have significantly less meiotic DSBs than organisms relying on DSBs including plants (Hunter 2015; Zickler and Kleckner 2016). Indeed, *C. elegans*, whose chromosome pairing is DSB-independent, exhibits a very small number of DSBs and only ZMM-dependent crossovers (Zickler and Kleckner 2015).

### Meiotic crossover control in brief

Why is the MUS81 endonuclease pathway not the major crossover pathway in plant meiosis? A partial answer to this question can be found in those eukaryotes in which crossover recombination is based entirely on MUS81. Fission yeast and several *Aspergillus* species do not have ZMM proteins, use DSB-independent chromosome pairing, lack canonical SC and crossover interference, and show crossover numbers per chromosome higher than those of ZMM-containing organisms (Zickler and Kleckner 1999; Auxier et al. 2022). This high number of crossovers is usually explained by the requirement for at least one crossover per chromosome pair, which can be ensured only by a relatively high CO frequency due to the random nature of Class II crossover placement (Wang et al. 2015b).

Undoubtedly, the key to the success of the ZMM pathway in dominating meiotic crossover recombination in most eukaryotes is its multi-level regulation. This regulation is partially SC-dependent: The transverse filament protein is required for Class I crossovers in many species, from budding yeast to mouse, implying that the SC has coevolved with ZMM proteins since the emergence of eukaryotes and that their functional separation is likely a recent development in plants (Pyatnitskaya et al. 2019; Capilla-Pérez et al. 2021). SC remains crucial for crossover assurance and interference, key factors for the most basic level of crossover regulation (Wang et al. 2015a; Capilla-Pérez et al. 2021; France et al. 2021; Durand et al. 2022). Natural variation in a number of SC and chromosome axis genes was shown to be important for plant adaptation by contributing to crossover number control (Yant et al. 2013; Wright et al. 2014; Dreissig et al. 2020). Other components of the ZMM pathway provide a much more precise mechanism for regulating the number of recombination events (Table 1). The E3 ligase HEI10 is a natural modifier of crossover frequency, showing great variability among Arabidopsis accessions. Increasing *HEI10* expression causes a linear increase in the number of Class I crossovers (Ziolkowski et al. 2017). Observations from high-resolution microscopy have led to the recent proposal of a diffusion-mediated coarsening model (Morgan et al. 2021; Zhang et al. 2021) in which large, evenly spaced HEI10 foci grow at the expense of smaller foci to define the final crossover sites (Morgan et al. 2021). This coarsening model has been recently supported by experimental data showing that SC is critical for funneling chromosomal HEI10 distribution (Durand et al. 2022) The dose-dependent effects of HEI10 on crossover number suggests the existence of factors that affect HEI10 expression and activity. A heat shock factor binding protein HSBP that regulates crossover number via controlling *HEI10* transcription has already been identified (Kim et al. 2022), and an additional protein functionally linked to HEI10, HEIP1, that participates in crossover regulation has been recently described in rice (Li et al. 2018). Moreover, other regulatory circuits, such as that including *HCR1*, which encodes a protein phosphatase, were shown to have anti-recombination roles in the regulation of the ZMM pathway (Nageswaran et al. 2021). All these mechanisms allow the fine-tuning of Class I crossovers (Table 1).

**Table 1:** Comparison of crossover control between ZMM- and MUS81-dependent pathways

Crossover pathway	Factor	Type of regulation	Reference in plant studies
ZMM (Class I Crossovers)	Synaptonemal complex (SC)	CO interference (increased distance between crossovers on the same chromosome)	(Wang et al. 2010, 2015a; Capilla-Pérez et al. 2021; France et al. 2021)
		CO assurance (at least one crossover per chromosome pair)	
		Negative control of CO number	
	HEI10 E3 ubiquitin or SUMO ligase	Smooth CO number adjustment	(Ziolkowski et al. 2017)
		CO interference (SC-mediated)	(Morgan et al. 2021)
	HSBP (HCR2)	Negative control of CO number (via repression of HEI10 expression)	(Kim et al. 2022)
	HCR1 (Protein phosphatase X1)	Negative control of CO number (by phosphorylation of MutS and/or MutL complexes)	(Nageswaran et al. 2021)
	HEIP1 (rice)	CO number control, mechanism unknown	(Li et al. 2018)
	MSH2 complexes	CO distribution control in response to DNA polymorphism	(Dong et al. 2002; Emmanuel et al. 2006; Ziolkowski et al. 2015; Blackwell et al. 2020; Serra et al. 2021)
MUS81-dependent (Class II crossovers)	FIGL1 AAA-ATPase together with its partner FLIP	Limiting Class II crossovers (via inhibiting strand invasion)	(Girard et al. 2015; Fernandes et al. 2018; Kumar et al. 2019)
	FANCM helicase complex (FANCM, MHF1, MHF2)	Limiting Class II crossovers (via JM dissociation)	(Crismani et al. 2012; Knoll et al. 2012; Girard et al. 2014; Fernandes et al. 2017; Blary et al. 2018; Mieulet et al. 2018)
	RECQ4 helicase complex (RECQ4A/B, TOP3α, and RMI1)	Limiting Class II crossovers (via JM dissociation)	(Séguéla-Arnaud et al. 2015, 2017; Mieulet et al. 2018)
	SMC5/6 complex (SNI1)	Limiting Class II crossovers (by affecting DNA helicase activity)	(Chen et al. 2021; Zhu et al. 2021)
	MSH2 complexes?	CO distribution control in response to DNA polymorphism?	

Regulation of the initial stages of meiotic recombination is not included. Question marks indicate potential uncharacterized factors regulating Class II crossover formation

While it has proven difficult to identify strong negative regulators limiting the formation of ZMM crossovers (outside of interference), many negative factors have been characterized for the MUS81-dependent crossover pathways. The major mechanisms in this case are based on the removal of different types of JMs that constitute potential MUS81 substrates by the DNA helicases FANCM and RECQ4 (Table 1) (Crismani et al. 2012; Knoll et al. 2012; Girard et al. 2014; Séguéla-Arnaud et al. 2015; Fernandes et al. 2017; Blary et al. 2018; Mieulet et al. 2018). Moreover, the regulation of the initial stages of strand invasion and D-loop formation by FIGLI AAA-ATPase and its interacting protein FLIP1 can affect MUS81-dependent crossover formation (and likely also the ZMM pathway, although this was not studied; Girard et al. 2015; Fernandes et al. 2018a). Recently, a component of the SMC5/6 complex, SNI1, has been shown to act as a natural modifier of MUS81-dependent crossovers in Arabidopsis (Zhu et al. 2021). SMC5/6 was demonstrated to affect JM formation, likely by controlling DMC1 loading (Chen et al. 2021; Zhu et al. 2021). Mutations in *SNI1* result in an increase in MUS81 crossovers, suggesting that this is another negative regulator of this crossover pathway (Zhu et al. 2021).

It should be emphasized that there is no coupling between the two crossover pathways that could indicate a mechanism for controlling the total crossover number: disabling ZMM crossover formation does not result in an increased number of MUS81-dependent crossovers (Higgins et al. 2004; Mercier et al. 2005; Falque et al. 2009). Also, increasing MUS81-dependent crossovers via disabling DNA helicases does not lead to a reduction of ZMM recombination (Crismani et al. 2012; Séguéla-Arnaud et al. 2015).

In summary, while the ZMM pathway in combination with SC provides a multi-level and smooth control of crossover frequency and chromosomal distribution, Class II crossovers appear to be mostly negatively regulated, at least in plants. While the MUS81 pathway has the potential to repair a huge number of DSBs through crossovers, the negative control has far-reaching consequences; it works more like an emergency brake that is difficult to adjust. Therefore, fine-tuning recombination with MUS81 would be like trying to carve the Venus de Milo using only a jackhammer.

### Recombination pathways and polymorphism sensitivity

At this point, however, it is worth asking an even more general question: what is the functional difference between crossover recombination in meiosis and recombination repair in somatic cells?

In somatic cells, the faithful repair of DNA damage is essential for the maintenance of genome integrity. Although a significant proportion of somatic DSBs are remediated by pathways that do not require a homologous template, especially non-homologous end joining, homologous recombination via MUS81 provides an alternative pathway in the repair of DSBs and interstrand cross-links (Hartung et al. 2006). MUS81-dependent recombination also plays an important role in the recovery of stalled replication forks. In these processes, the repair substrate should be identical to the damaged DNA molecule.

However, substrate choice is completely different in meiotic crossover recombination, the basis of which is the mutual exchange of fragments between homologous chromosomes, i.e., material from both parents. In many cases, the homologous chromosomes are not identical but differ at the nucleotide sequence level. Therefore, the meiotic crossover must to some extent allow for interhomolog polymorphisms between the recombining chromosomes. Moreover, differences at the sequence level may be useful for distinguishing between sister and homolog chromatids, although interhomolog bias is performed in part by DMC1 recombinase (Kurzbauer et al. 2012; Pradillo et al. 2012; Lee et al. 2015). To what extent are these meiotic requirements fulfilled by MUS81 and ZMM crossover pathways, respectively?

Studies in *A. thaliana* suggest that the MUS81-dependent crossovers show a relative reluctance to interhomolog polymorphisms compared to the ZMM pathway. For example, in *recq4* mutants, where MUS81-dependent crossover repair is unleashed, a negative correlation between the recombination frequency of a given interval and its polymorphism level is observed (Fernandes et al. 2017). At the kilobase scale, an analysis of SNP density around crossover midpoints showed that ﻿crossovers are positively associated with interhomolog polymorphisms in wild-type Arabidopsis but not in *recq4* mutants (Blackwell et al. 2020). Even more extreme differences are seen in the Arabidopsis *fancm* mutant, which also boosts MUS81-dependent crossover repair; while a significant increase in crossover frequency is observed in inbred plants, there is practically no change in recombination frequency in hybrid Arabidopsis plants (Crismani et al. 2012; Fernandes et al. 2017). These observations illustrate the differences between the substrates processed by RECQ4 and FANCM but also show how these two DNA helicases affect the MUS81 ability to create crossovers in polymorphic regions.

However, all these observations need to be considered with caution, as there are based entirely on data from F_1_ hybrids where it is very difficult to detangle the polymorphism effect from the chromosomal position effect, and the two are strongly correlated due to historical level of recombination (Kim et al. 2007; Andersen et al. 2012; Charlesworth and Campos 2014). To directly investigate the role of interhomolog polymorphism on the crossover formation, it is necessary to use an experimental system in which epigenetic effects or those related to chromosomal localization will be eliminated. Such a system was developed by obtaining *A. thaliana* lines that differ in the combination of homozygous and heterozygous regions on the same chromosome. Experiments carried out using these lines in the *fancm zip4* background, where only Class II crossover pathway is active, showed that crossovers are formed mostly in homozygous regions (Ziolkowski et al. 2015). This further suggests decreased ability of MUS81 to act in regions with elevated polymorphism level.

In the case of the ZMM pathway, enlistment of the MSH4-MSH5 heterodimers, components of the MutS mismatch repair protein family, may suggest that the tolerance of DNA polymorphism or sensing of mismatches between recombination substrates could be important in establishment of this pathway. Both MSH4 and MSH5 lack one of two domains that are essential for binding to mismatches, which presumably allows a complex to be tolerant for mismatch-containing heteroduplexes (Manhart and Alani 2016). Additionally, the components of the MutLγ heterodimer (MLH1-MLH3), the major resolvase of the ZMM pathway, are derived from the MutL endonuclease family, which is also involved in MMR. It is therefore tempting to speculate that the ZMM pathway has emerged to cope with imperfectly matched sequences as substrates for recombination.

Indeed, the ZMM crossover pathway shows tolerance and even to some extent preference for polymorphic regions in Arabidopsis. A study of crossover distribution at the kilobase scale in various *A. thaliana* hybrids showed a parabolic relationship between SNP density and crossover rate; that is, an initial positive correlation between crossover occurrence with increasing SNP density turns into a negative correlation beyond a certain threshold (Blackwell et al. 2020). However, a recent analysis comparing the chromosomal distribution of crossovers between hybrid and inbred lines showed no significant difference at the chromosomal scale (Lian et al. 2022). This indicates that crossover distribution is largely shaped by factors other than the presence or absence of polymorphism (Choi et al. 2013, 2018; Hsu et al. 2021; Lian et al. 2022). Identifying the effect of interhomolog polymorphism again requires the use of lines that differ only in the pattern of heterozygosity (Ziolkowski et al. 2015). Experiments carried out using these lines revealed a chromosomal crossover distribution remodeling in response to the pattern of heterozygosity: it has been shown that wild-type Arabidopsis shifts crossovers from homozygous (identical in both parents) to heterozygous (differing between parents) chromosomal regions. This homozygosity–heterozygosity juxtaposition effect seems to be interference-dependent and specific to the ZMM pathway only (Ziolkowski et al. 2015; Blackwell et al. 2020). It appears that it could not function without the strict control of crossover distribution that only the ZMM pathway provides (Ziolkowski and Henderson 2017). Interestingly, slightly though significantly increased recombination was recently reported in heterozygous regions of maize plants selfed for six generations, which may indicate that this phenomenon also occurs in other angiosperms (Roessler et al. 2019). Thus, the ZMM pathway provides a toolkit to shape the crossover distribution in response to the pattern of heterozygosity.

As already mentioned, the key components of the ZMM pathway are derived from the MMR system, although they have probably lost their mismatch detection capability (Manhart and Alani 2016). Apart from the meiosis-specific MSH4-MSH5 complex, other complexes of MutS homologs are also active during the prophase of meiosis I, influencing crossover formation. In budding yeast, these complexes invariably block crossovers in polymorphic regions (Borts and Haber 1987; Chen and Jinks-Robertson 1999; Cooper et al. 2021). On the contrary, Arabidopsis F_1_ hybrids in the *msh2* mutant background, where MMR complexes responsible for mismatch detection are disabled, show the redistribution of crossovers from the diverse pericentromeres to regions of lower variability (Blackwell et al. 2020). Moreover, the heterozygosity–homozygosity juxtaposition effect disappears in *msh2*, and the opposite although much weaker phenomenon is observed, with crossover remodeling from heterozygous to homozygous regions (Blackwell et al. 2020). These observations suggest that the ability of the ZMM pathway to form crossovers in polymorphic regions is determined by the unexpected pro-recombinational activity of the MMR elements. How could MMR have an opposite effect on meiotic recombination in budding yeast and Arabidopsis? Perhaps this is a consequence of the fact that ZMM does not strictly require MMR for crossover formation (MMR is likely not part of the ZMM pathway), which makes MMR useful for the control of recombination, locally stimulating or suppressing it in response to polymorphism as needed in different groups of organisms.

### Crossover distribution from the population genetics perspective

Why would it be beneficial for plants to target recombination to polymorphic regions? The answer may lie in the reproduction strategy adopted by many plant species: selfing. Despite the well-characterized benefits of outcrossing, it is estimated that approximately 20% of angiosperms have evolved selfing as the predominant reproductive strategy. At least one-third of all flowering plant species adopt a mixed mating strategy, with both selfing and outcrossing (Vogler and Kalisz 2001; Barrett 2002). Selfing is considered to be favored because of its inherent transmission advantage as well as the benefit of ensuring reproduction when pollinators or mates (or both) are scarce (Stebbins 1957; Charlesworth and Wright 2001).

In inbreeding populations, meiotic recombination occurs between closely related genomes with high frequencies of homozygote loci. Thus, the effective recombination rate between polymorphic sites is low (Charlesworth and Wright 2001). In extreme cases, it resembles the conditions in asexually reproducing organisms, despite the occurrence of meiosis-related random chromosome segregation and crossover (Nordborg 2000). As a consequence of the reduced effective population size and selective interference among linked sites, selfing is associated with various long-term costs, including inbreeding depression, genetically uniform populations, greater population subdivision, and more frequent genetic bottlenecks (Charlesworth and Wright 2001; Wright et al. 2013). These properties of selfing lineages have the potential to significantly increase the rates of extinction (Lynch et al. 1995; Wright et al. 2013).

**Fig. 2:**
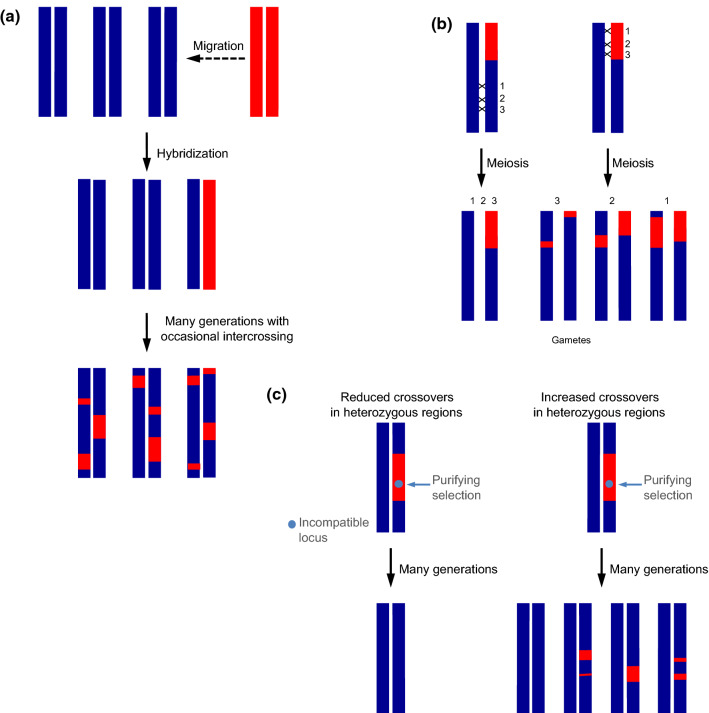
The relationship between recombination and genome evolution in a highly inbreeding population. For simplicity, only one chromosome pair was shown for an individual. **a** Occasional outcrossing with a migrant from a diverged population (red) to an inbred population (blue), followed by intercrossing within the inbred population, on a short time scale produces mosaic genomes composed of heterozygous fragments surrounded by homozygous blocks. In selfing populations, there will be a further loss of heterozygosity over time but with retention of some of the migrant’s genetic background (not shown). **b** The genetic consequences of crossover distribution depend on whether it is located in a homozygous or heterozygous region. Crossover placement in a homozygous region results in the reconstruction of parental genotypes regardless of the location of the event (left), while placement in heterozygous regions leads to the generation of a new allele pattern, with each event resulting in the production of a different pattern of alleles in the gametes, always different from the parental pattern (right). Blue and red bars represent genetic material with different ancestry. Scenarios for three meioses with one crossover per each meiosis (marked ‘X’ and numbered) are shown. The gametes resulting from all three scenarios are shown below. **c** Increased crossover recombination in heterozygous regions enables uncoupling neutral alleles from the migrant parent (red) from BDMIs (gray circle). Increased recombination permits the maintenance of variation that would otherwise be lost to purifying selection due to low recombination

Even highly inbreeding organisms occasionally outcross; for example, self-fertilized *Arabidopsis thaliana* has a 2–3% average outcrossing rate in nature (Abbott and Gomes 1989). Outcrossing can occur when two populations that have evolved independently for some time meet again. Although this may occur as a result of the disappearance of the barrier that originally separated the populations (e.g., geographic isolation), in plants, it is more likely to occur when seeds or pollen are accidentally transferred between separated inbreeding populations. As a consequence, a single individual with a diverged genotype will emerge in an almost homozygous population. Occasional interbreeding between this individual and other plants within the population will lead to the gradual elimination of the migrant (= divergent) genetic background from the population over time. This leads to the formation of a mosaic genome structure in which heterozygous chromosomal blocks are flanked by homozygous regions (Fig. 2A). Crossover placement in homozygous regions does not generate new population variability and therefore does not contribute to the effective population size (Fig. 2B). On the other hand, recombination in heterogeneous regions leads to the generation of new allelic combinations in the offspring (Fig. 2B). This can be beneficial for organisms with reduced adaptation to their environment (Henderson and Bomblies 2021). For example, the migrant can bring both beneficial and disadvantageous alleles to the inbreeding population, thus targeting recombination to the newly formed heterozygous regions, consisting of both migrant and non-migrant DNA, allow to eliminate these suboptimal combinations by breaking their linkage.

Increasing the frequency of recombination in polymorphic chromosomal regions would also make sense in light of Bateson–Dobzhansky–Muller incompatibility (BDMI). While BDMI has traditionally been used to describe the processes involved in speciation (Bomblies et al. 2007; Masly and Presgraves 2007), it has recently been applied successfully to explain the evolution of hybrid genomes (Schumer et al. 2018). According to this model, introgression from a lineage with a smaller effective population size may produce hybrids that suffer from the introduction of weakly deleterious alleles (Bierne et al. 2002). One of the properties of meiotic recombination is its ability to uncouple linked variants, which increases the efficiency of selection. Indeed, the BDMI models and experimental data show that neutral alleles from the migrant parent are more likely to persist in chromosomal regions with a higher crossover frequency, where uncoupling from deleterious polymorphisms in the genetic background of the prevalent parent is faster (Fig. 2C) (Schumer et al. 2018). Thus, the acquisition of a mechanism to increase the crossover frequency in heterozygous regions would permit more rapid elimination of BDMIs from the population while maintaining neutral migrant genetic material.

### Future perspectives

Despite recent progress in our understanding of the relationship between polymorphisms and recombination, many questions remain unanswered. One problem lies in the limitations of our experimental systems. For example, when studying the effects of mutations in individual genes involved in recombination, we are often doomed to compare effects in the mutant vs. a wild-type control. In such cases, as a consequence of crossover interference, there is often a remodeling of crossover distribution along the chromosomes, which can significantly hinder the interpretation of the results at the local scale. The solution to this problem could be to use experimental systems based on lines with different patterns of heterozygosity. The development of such systems would be of particular interest at the kilobase scale.

Recent reports from wheat identifying the *Ph2* locus as a gene encoding MSH7, a plant-specific paralog of MSH6, show that there is still much to be discovered regarding the importance of the MMR system in the formation of meiotic crossovers in plants (Dong et al. 2002; Serra et al. 2021). Systematic analysis of mutants for other genes in the MutS complexes could provide new insights. The molecular mechanism by which MMR affects crossover formation is unknown but could potentially be elucidated using high-resolution microscopy. There are also unanswered questions about the impacts of MMR proteins on the activity of meiotic repair pathways other than the ZMM pathway. Differences in the activities of these pathways observed between *fancm* and *recq4* mutants in inbred and hybrid contexts suggest that the activity of the DNA helicases and MUS81 can be affected by MMR (Girard et al. 2015; Ziolkowski et al. 2015; Fernandes et al. 2017).

The considerations presented in this opinion are largely based on research carried out in *A. thaliana*, which possesses an exceptionally simple genome relative to the complex, repeat-rich genomes of many other plant species. These differences impact, among other phenomena, the chromosomal crossover distribution. For example, in many crops, the vast majority of crossovers occur in relatively nonpolymorphic subtelomeric regions, while the remainder of the chromosome, especially the highly polymorphic pericentromeric regions, remains inaccessible to recombination (Choulet et al. 2014; Li et al. 2015; Mascher et al. 2017). This is probably because other genetic and epigenetic factors have a greater impact on the crossover distribution (Rodgers-Melnick et al. 2015; Blackwell et al. 2020; Hsu et al. 2021). Moreover, there are differences in the polymorphism sensitivity of particular repair pathways between different species. For example, while the *fancm* mutation is unable to increase the crossover frequency in *A. thaliana* hybrids and closely related *Brassica napus* allohaploids, an approximately twofold increase is seen when the gene is turned off in rice and pea (Girard et al. 2015; Ziolkowski et al. 2015; Fernandes et al. 2017; Blary et al. 2018; Mieulet et al. 2018). Therefore, to understand to what extent the relationship between polymorphism and crossover is universal among plants, it will be necessary to conduct similar studies in other species. In this context, several points remain to be addressed: To what extent do different reproduction strategies (selfing vs. outcrossing) and plant genome organization features (e.g., genome size, TE content) translate into the relationship between interhomolog polymorphism and crossover placement? Is crossover targeting heterozygous regions unique to plants with simple genomes and/or self-pollinating behavior, such as Arabidopsis? Can the heterozygosity pattern in which heterozygous regions are adjacent to homozygous regions efficiently shape crossover placement while overcoming the effects caused by other genetic and epigenetic features? To what extent does crossover remodeling by polymorphisms affect population variability and evolutionary processes over generations? Answering these questions will not only broaden our understanding of meiotic recombination control and its importance for plant evolution but also open up new perspectives in plant breeding, for example, by enabling crossover-based gene transfer between distant species.
